# Marine Bromophenol Bis(2,3,6-Tribromo-4,5-Dihydroxybenzyl)ether Inhibits Angiogenesis in Human Umbilical Vein Endothelial Cells and Reduces Vasculogenic Mimicry in Human Lung Cancer A549 Cells

**DOI:** 10.3390/md19110641

**Published:** 2021-11-16

**Authors:** Songtao Dong, Zhongyuan Chen, Li Wang, Yankai Liu, Dimitrios Stagos, Xiukun Lin, Ming Liu

**Affiliations:** 1 Key Laboratory of Marine Drugs, Ministry of Education of China, School of Medicine and Pharmacy, Ocean University of China, 5 Yushan Road, Qingdao 266003, China; 21190831116@stu.ouc.edu.cn (S.D.); 21190831021@stu.ouc.edu.cn (Z.C.); 21190811062@stu.ouc.edu.cn (L.W.); liuyankai@ouc.edu.cn (Y.L.); 2 Laboratory for Marine Drugs and Bioproducts of Qingdao National Laboratory for Marine Science and Technology, Qingdao 266237, China; 3 Department of Biochemistry and Biotechnology, School of Health Sciences, University of Thessaly, Biopolis, 41500 Larissa, Greece; stagkos@med.uth.gr; 4 Department of Pharmacology, School of Pharmacy, Southwest Medical University, 319 Zhongshan Road, Jiangyang, Luzhou 646000, China; xiukunlin@swmu.edu.cn

**Keywords:** anti-angiogenesis, bromophenols, tube formation, vasculogenic mimicry

## Abstract

Angiogenesis, including the growth of new capillary blood vessels from existing ones and the malignant tumors cells formed vasculogenic mimicry, is quite important for the tumor metastasis. Anti-angiogenesis is one of the significant therapies in tumor treatment, while the clinical angiogenesis inhibitors usually exhibit endothelial cells dysfunction and drug resistance. Bis(2,3,6-tribromo-4,5-dihydroxybenzyl)ether (BTDE), a marine algae-derived bromophenol compound, has shown various biological activities, however, its anti-angiogenesis function remains unknown. The present study illustrated that BTDE had anti-angiogenesis effect in vitro through inhibiting human umbilical vein endothelial cells migration, invasion, tube formation, and the activity of matrix metalloproteinases 9 (MMP9), and in vivo BTDE also blocked intersegmental vessel formation in zebrafish embryos. Moreover, BTDE inhibited the migration, invasion, and vasculogenic mimicry formation of lung cancer cell A549. All these results indicated that BTDE could be used as a potential candidate in anti-angiogenesis for the treatment of cancer.

## 1. Introduction

Angiogenesis, the growth of new capillary blood vessels from existing ones and capillary venules, involves vascular endothelial cell proliferation, migration, matrix degradation, and branching to form new tubes [1]. It has been recognized as a proven sign in tumor growth and metastasis on account of the functional blood supply [2]. Therefore, targeting angiogenesis is a valid strategy for tumor treatment [3]. In recent years, anti-angiogenic agents have been used clinically [4,5]. For example, bevacizumab, the recombinant humanized monoclonal antibody, playing obvious anti-angiogenesis effect, has been used clinically to treat various malignant tumors through binding with VEGF [6]. Another anti-tumor drug ENDOSTAR, inhibits cancer angiogenesis through targeting vascular EGFR, has been used in clinical tumor treatment [7]. However, these anti-angiogenesis agents usually bring about endothelial cells dysfunction and exhibit drug resistance [8]. Safer and more valid approaches and agents in anti-tumor angiogenesis are required.

Besides the classical angiogenesis, Maniotis et al. firstly propose the concept of vasculogenic mimicry, which is a spontaneous and endothelial cell-independent tube-forming procedure [9]. Vasculogenic mimicry is regarded as an important blood supply system in tumor development for providing nutrients and oxygen [10]. Vasculogenic mimicry is an alternative angiogenesis happened to metastatic and aggressive tumors such as pancreatic cancer [11], melanoma [12], breast cancer [13], and non-small cell lung cancer (NSCLC) [14]. When vasculogenic mimicry occurs, tumor cells have significant extent of plasticity [15] and epithelial-mesenchymal transition (EMT) process [16]. Moreover, various extracellular matrix remodeling factors such as hypoxia inducible factor 1 alpha (HIF-1α) and vascular endothelial cadherin (VE-cadherin) are involved in these processes. The powerful metastasis ability of lung cancer accounts for high incidence and mortality, and vasculogenic mimicry not only leads to lung cancer metastasis but also increases the difficulty of anti-angiogenesis treatment [17]. Therefore, inhibitors targeting both endothelial angiogenesis and vasculogenic mimicry will be a new strategy in the treatment of NSCLC.

**Figure 1:**
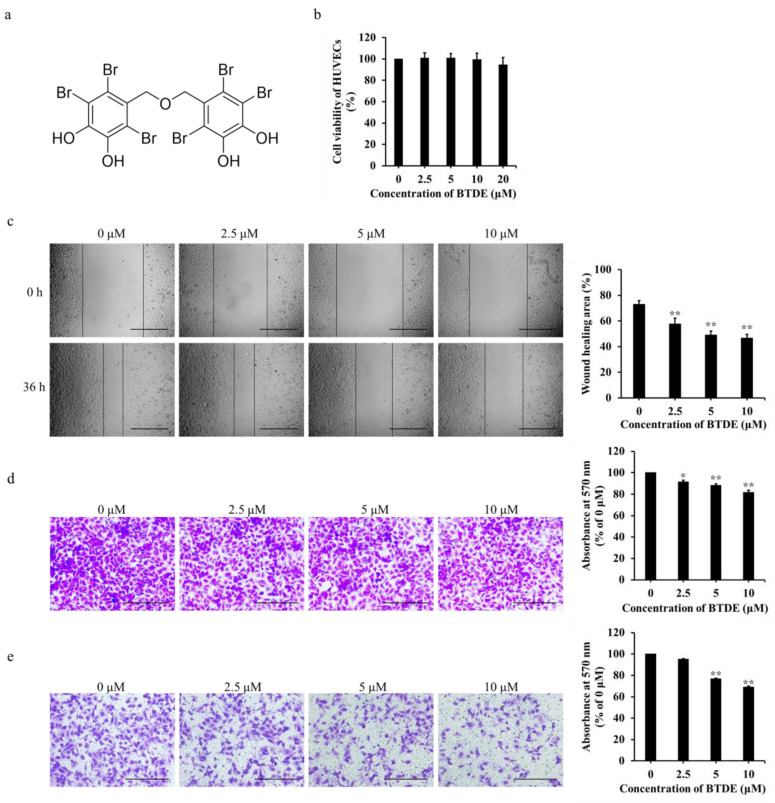
Bis(2,3,6-tribromo-4,5-dihydroxybenzyl)ether (BTDE) inhibits the migration and invasion of HUVECs. (**a**) Chemical structure of BTDE. (**b**) HUVECs was incubated in absence or presence of certain concentrations of BTDE at 37 °C for 36 h, cell viability was determined by MTT assay. (**c**) Wound healing of HUVECs after 36 h treatment with BTDE was reported by inverted microscope (original magnification, 4×; scale bar: 600 μm) and the wound-healing area was measured by Image J software. Migration (**d**) and invasion (**e**) abilities of HUVECs were examined by transwell assay. Photos of HUVECs traveled through membrane after incubation with BTDE for 24 h were recorded by inverted microscope (original magnification, 10×; scale bar: 300 μm) and OD values at 570 nm were measured. Data are represented as mean ± SD of three independent experiments. * *p* < 0.05, ** *p* < 0.01 versus control.

Marine compounds are reported to have anticancer therapeutic and prophylactic activities [18,19,20,21], among them, marine bromophenols mainly distributing in the algae have attracted much attention in function[nal food and pharmaceutical drugs area. Previous studies have shown that bromophenols have a variety of biological activities, such as anti-tumor, anti-oxidation, anti-diabetic, and anti-viral activities [22,23]. Interestingly, the ability of bromophenols in anti-angiogenesis has also been widely reported. For example, BDDPM, a bromophenol from marine red alga *Rhodomela confervoides*, shows anti-angiogenesis properties by targeting multiple receptor tyrosine kinases [24]. Another bromophenol compound BDDE, obtained from *L. nana and Rhodomela confervoides*, exhibits anti-angiogenesis effect both in vivo and in vitro through acting on VEGF signaling pathway [25]. Bis(2,3,6-tribromo-4,5-dihydroxybenzyl)ether (BTDE, Figure 1a), a typical bromophenol compound first derived from marine red alga *Symphyocladia latiuscula* [26], has a variety of biological activities, such as antioxidant [27,28], antidiabetic [29], anti-neurodegenerative diseases [30], and multiple enzyme inhibitory activity [31,32]. However, its effects in tumor angiogenesis have yet to be illustrated. In the present study, in order to investigate the anti-angiogenesis activity of BTDE both in vitro and in vivo, we evaluated the effects of BTDE on the migration, invasion, tube formation, and matrix metalloproteinases 9 (MMP9) activity on HUVECs model, and also on the growth of intersegmental blood vessel (ISV) in vivo using zebrafish embryos model. Furthermore, the effect of BTDE on the vasculogenic mimicry formation ability of A549 cells was also estimated.

## 2. Results

### 2.1. BTDE Inhibits the Migration and Invasion of HUVECs

HUVECs is widely used in vitro to detect the ability of angiogenesis. MTT assay was applied first to measure the effect of BTDE on HUVECs proliferation. As shown in Figure 1b, BTDE had no cytotoxicity effect on HUVECs at 2.5−20 μM concentrations, indicating BTDE could not affect the proliferation of HUVECs under these experimental conditions. Endothelial cells migration is one of the crucial steps in blood vessels formation. To investigate the influence of BTDE on HUVECs migration, scratch-wound cell migration assay and transwell migration assay were used. As shown in Figure 1c, the migration area of HUVECs was inhibited after 36 h treatment by 2.5−10 μM BTDE with the wound healing percentage of 57.6, 49.1, and 46.8%. Moreover, in the transwell migration assay, the number of HUVECs traveling through the membrane was significantly reduced with the increased concentrations of BTDE (Figure 1d). Similarly, endothelial cells invasion is a pivotal step promoting HUVECs migration and neovascularization through degrading extracellular matrix [33]. Transwell invasion assay was used to investigate the invasion ability of HUVECs, and as shown in Figure 1e, the number of HUVECs degrading matrigel and traveling through the membrane was decreased with the treatment of BTDE. The above results proved that BTDE could inhibit the migration and invasion of HUVECs.

### 2.2. BTDE Reduces HUVECs Tube Formation and MMP9 Activity

**Figure 2:**
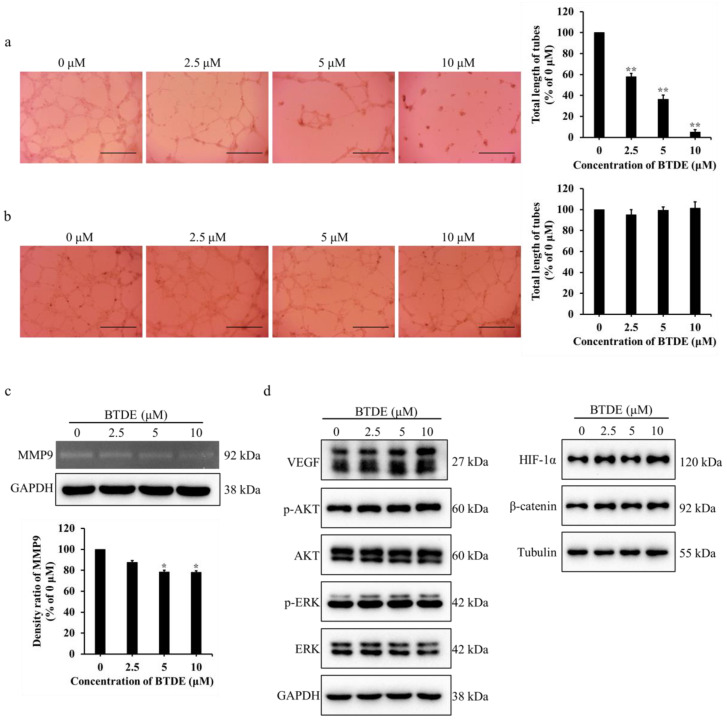
BTDE reduces HUVECs tube formation and MMP9 activity. (**a**) HUVECs was pretreated with BTDE for 24 h, then seeded on matrigel for 20 h, capillary-like structures of HUVECs were recorded by inverted microscope (original magnification, 4×; scale bar: 600 μm) and total length of tubes was measured by Image J software. (**b**) Different concentrations of BTDE were added after tubes have established on matrigel for 8 h, and incubated for another 6 h. Tubular structures were observed by inverted microscope (original magnification, 4×; scale bar: 600 μm) and total length of tubes compared with 0 μM was measured by Image J software. (**c**) Gelatin zymography experiment was used to detect the MMP9 activity of HUVECs after 24 h treatment of BTDE, GAPDH was used as an internal control. (**d**) Western blot was used to measure the VEGF, HIF-1α, β-catenin, AKT, and ERK as well as their phosphorylation levels in HUVECs treated with BTDE for 24 h. Data represent mean ± SD of three independent experiments. * *p* < 0.05, ** *p* < 0.01 versus control.

Tube formation assay is a valid method to examine the effect of angiogenesis using matrigel to simulate endothelial cell growth and tube formation in vitro [34]. To further evaluate the effect of BTDE on vessel formation, tube formation assay was used with or without BTDE treatment on matrigel. As shown in Figure 2a, the endothelial tubes were significantly decreased and the total length of tubes dropped to 58.1, 36.3, and 4.9% when treated with 2.5−10 μM BTDE. These results illustrated that BTDE could restrain the tube formation of HUVECs. To further investigate whether BTDE has an impact on preformed vascular tubes, different concentrations of BTDE were added after tubes had already formed for 8 h, and incubated for another 6 h. The result showed that BTDE had no effect on the preformed tubes (Figure 2b). The above results exhibited that BTDE inhibited the tube formation but not the preformed vascular tubes of HUVECs.

MMPs are the important enzymes secreted by cells to degrade the extracellular matrix, and they play a significant role in endothelial cells migration, invasion, and angiogenesis [35,36]. Our results have confirmed that BTDE inhibited HUVECs migration, invasion, and tube formation, to further explore whether BTDE affects the activity of MMPs in HUVECs, gelatin zymography assay was used. HUVECs culture medium treated with different concentrations of BTDE were separated by SDS-PAGE containing gelatin, and incubated for 48 h. As shown in Figure 2c, BTDE inhibited the activity of MMP9 in HUVECs compared with control group which had obvious negative staining bands.

VEGF is a crucial pro-angiogenic factor which plays an important role in promoting tumor angiogenesis, moreover, AKT and ERK as its downstream signaling molecules participate in the regulation of angiogenesis [37,38,39]. HIF-1α as a significant transcriptional factor acts on Wnt/β-catenin pathway and regulates expression of genes that promote angiogenesis such as VEGF [40]. Therefore, we examined whether BTDE influences these molecules. As shown in Figure 2d, BTDE did not affect expression level of VEGF, HIF-1α, β-catenin, AKT, ERK, as well as the phosphorylation levels of AKT and ERK in HUVECs. The above experiments indicated that BTDE inhibits HUVECs tube formation and MMP9 activity, while did not affect the VEGF, HIF-1α, β-catenin expression.

### 2.3. BTDE Blocks Intersegmental Vessel Formation in Zebrafish Embryos

**Figure 3:**
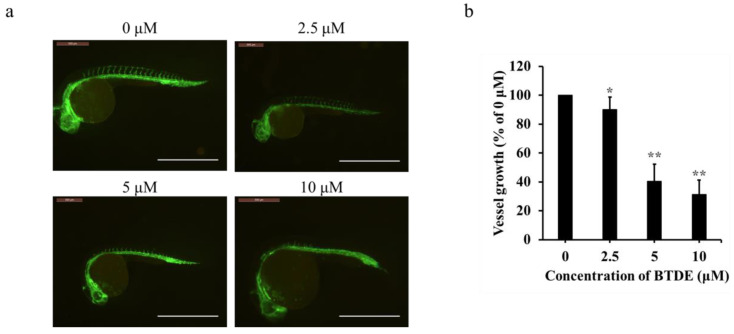
BTDE blocks intersegmental vessel formation in zebrafish embryos. (**a**) Lateral view of Tg (flk1: EGFP) zebrafish embryos at 16 hpf. Embryos were treated with different concentrations of BTDE. Vessels of zebrafish embryos were observed using a fluorescence microscope, photos were recorded by inverted fluorescence microscope (scale bar: 1.2 mm). (**b**) Quantification of intersegmental vessel growth induced by BTDE. Values represent the means ± SD of three independent experiments. * *p* < 0.05, ** *p* < 0.01 versus medium control.

Zebrafish is an ideal model for evaluating the effects of compounds on angiogenesis. It can sprout from dorsal arteries to form interstitial neovascularization during embryonic development [41,42]. To further confirm the anti-angiogenesis effect of BTDE in vivo, the formation of ISV in zebrafish embryos was detected. As shown in Figure 3a and b, ISV formation in zebrafish embryos was significantly suppressed by 2.5−10 μM BTDE with the vessel growth of 90.1, 40.3, and 31.2%, respectively, illustrating that BTDE exerted well anti-angiogenesis effect in vivo. Besides, zebrafish toxicity assay showed that 2.5−20 μM BTDE had no specific deformity and mortality effects on zebrafish embryos (Table 1), which indicated that BTDE was nontoxic at these concentrations.

### 2.4. BTDE Decreases the Migration and Invasion of A549

**Figure 4:**
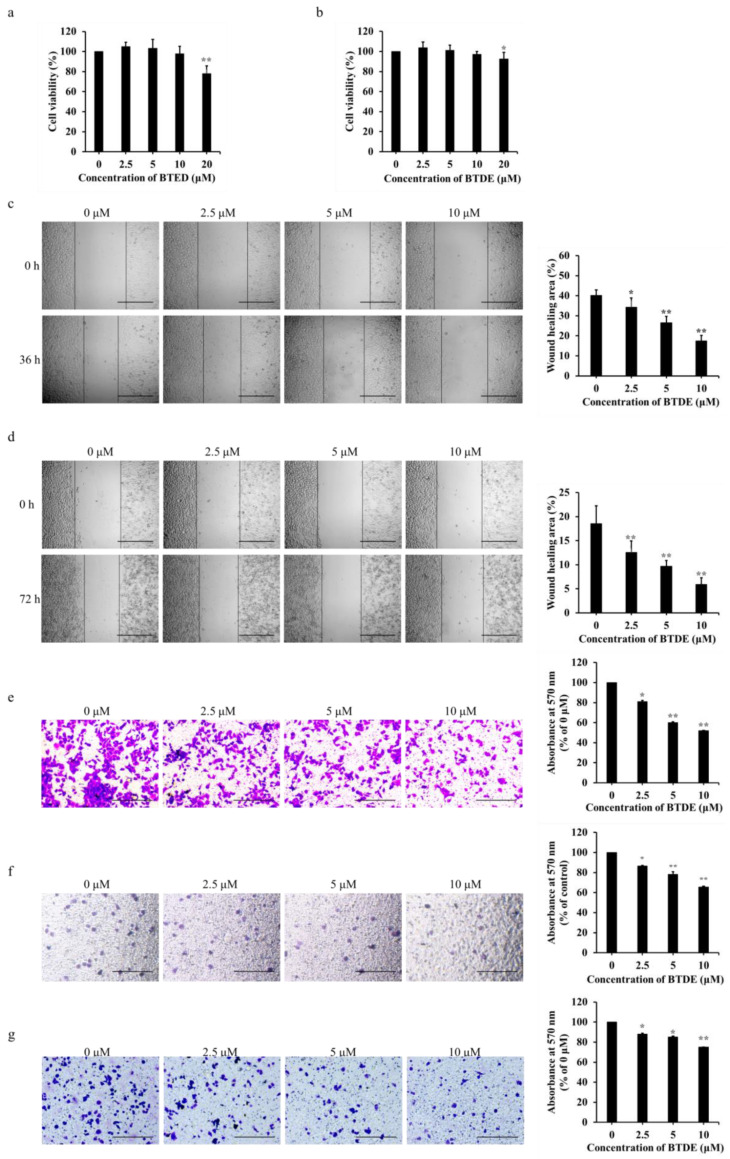
BTDE decreases the migration of A549, H1975 and the invasion of A549. The effect of BTDE on A549 (**a**) or H1975 (**b**) proliferation. A549 or H1975 was incubated with different concentrations of BTDE at 37 °C for 48 h, cell viability was determined by MTT assay. Wound healing of A549 after 36 h (**c**) or H1975 after 72 h (**d**) treatment with 0−10 μM BTDE was recorded with inverted microscope (original magnification, 4×; scale bar: 600 μm) and the wound healing area was measured by Image J software. Migration ability of A549 (**e**) or H1975 (**f**), invasion ability of A549 (**g**) treated with 0−10 μM BTDE for 24 h, photos were obtained by inverted microscope (original magnification, 10×; scale bar: 300 μm) and OD values of 570 nm were measured. Data are represented as mean ± SD of three independent experiments. * *p* < 0.05, ** *p* < 0.01 versus control.

In addition to study whether BTDE inhibited the endothelial angiogenesis, we next investigated the impact of BTDE on NSCLC vasculogenic mimicry. First, MTT assay related that 2.5−10 μM BTDE had no cytotoxicity effect on A549 and H1975 cells (Figure 4a,b). Considering migration of cancer cells are important for the formation of vasculogenic mimicry, we then detected the effects of BTDE on migration abilities of A549 and H1975 by using scratch-wound cell migration assay. Results showed that BTDE inhibited A549 migration with wound-healing area of 34.2, 26.5, and 17.5% under 36 h treatment of 2.5−10 μM BTDE (Figure 4c), and also restrained H1975 migration with wound-healing area of 12.6, 9.7, 5.9% with 72 h treatment of 2.5−10 μM BTDE (Figure 4d). Moreover, the number of A549 and H1975 migrated to the lower membrane was decreased with the increasing concentration of BTDE (Figure 4e,f). Transwell invasion assay illustrated that BTDE suppressed A549 to split matrigel and migrate to the lower surface of membrane (Figure 4g). The above results exhibited that BTDE inhibited A549, H1975 migration and invasion of A549.

### 2.5. BTDE Decreases the Vasculogenic Mimicry of A549 Cells

**Figure 5:**
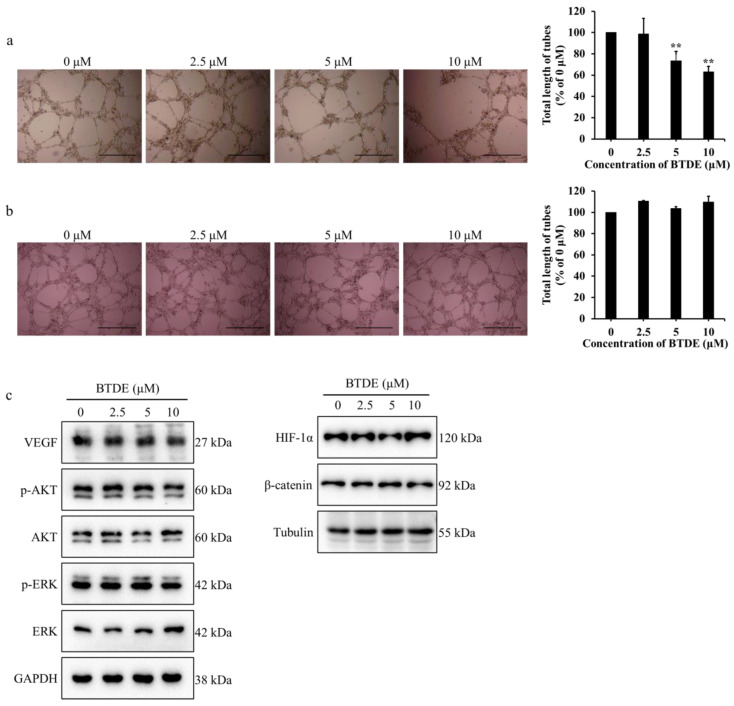
BTDE decreases the vasculogenic mimicry of A549 cells. (**a**) A549 was pretreated with BTDE for 24 h, then seeded on matrigel for 30 h, capillary-like structures of A549 were recorded by inverted microscope (original magnification, 4×; scale bar: 600 μm) and total length of tubes was measured by Image J software. ** *p* < 0.01 versus control. (**b**) Different concentrations of BTDE were added after tubes were established on matrigel for 6 h, and incubated for another 20 h. Tubular structures were observed by inverted microscope (original magnification, 4×; scale bar: 600 μm) and total length of tubes compared with 0 μM was measured by Image J software. (**c**) Western blot was used to measure the VEGF, HIF-1α, β-catenin, AKT, and ERK as well as their phosphorylation levels in A549 treated with BTDE for 24 h. Data are represented as mean ± SD of three independent experiments. ** *p* < 0.01 versus control.

Vasculogenic mimicry is the endothelial cell independent vascularization pretend the plasticity of tumor cells. In the above study, we have already proved that BTDE inhibited the migration of NSCLC cells, considering the stronger migration ability of A549 cells, we next explored if BTDE affects A549 vasculogenic mimicry formation ability using vasculogenic mimicry assay. A549 was pretreated with different concentrations of BTDE for 24 h and then seeded on matrigel for 30 h. As shown in Figure 5a, the control group formed reticular vessel-like structures while 5 and 10 μM BTDE treated group formed a loose network structure with total length of tubes dropped to 73.3 and 63.1%. To further evaluate whether BTDE had an impact on the preformed vascular tubes, different concentrations of BTDE were added after tubes had already formed for 6 h, and incubated for another 20 h. The result showed that BTDE had no effect on the preformed tubes compared with control group (Figure 5b). These results illustrated that BTDE could inhibit the vasculogenic mimicry formation ability of A549 while did not affect the preformed vessels. To further explore the specific mechanism of BTDE inhibiting vasculogenic mimicry formation of A549, Western blot assay was used to detect the influence of BTDE on HIF-1α, β-catenin, VEGF, and its downstream AKT, ERK signaling pathways. We found that BTDE did not affect the expression of these molecules in A549 (Figure 5c). This was different from the previous study that bromophenol BOS-102 exhibited valid cytotoxic effects on A549 through ROS-mediated inhibition of PI3K/Akt and activation of p38/ERK signaling pathways [43], which may be caused by the difference of molecular structure and suggesting a novel mechanism. These results indicated that BTDE inhibited the vascular mimicry formation of A549 but had no effect on the preformed blood vessels of A549.

## 3. Discussion

Endothelial cells-mediated angiogenesis has been considered as a crucial process in angiogenesis because of the powerful abilities to migrate, invade, and degrade extracellular matrix to form new blood vessels [1]. Blood vessels play an important role in material exchange and pathological angiogenesis becomes a significant factor of cancer, therefore anti-angiogenesis is a common adjuvant strategy in tumor treatment [2]. Seeking novel drug candidates from natural products especially from marine resources has been implemented for many years, a number of marine bromophenols with significant anti-angiogenesis activity were found such as BDDPM [24] and BDDE [25]. Marine bromophenol BTDE illustrated various bioactivities including antioxidant [27] and antidiabetic [29], however, its anti-angiogenesis effect has not been explored. In the present study, we demonstrated first that BTDE potentially inhibited angiogenesis both in vitro and in vivo, and could be used as a promising candidate in cancer therapy. BTDE suppressed multiple angiogenesis process in endothelial cells, including the migration, invasion, and tube formation, which were consistent with some anti-angiogenesis drugs used clinically such as Bevacizumab [44]. BTDE showed no cytotoxicity on HUVECs proliferation in a short period, suggesting that the ability of BTDE to reduce HUVECs movement and angiogenesis did not include the influence on its proliferation. The in vivo zebrafish embryos assay also proved the anti-angiogenic effect of BTDE. MMPs are important enzymes secreted by endothelial cells, which promotes the cells migration and sprout to form new blood vessels by degrading extracellular matrix [36]. We found that BTDE indeed inhibited the activity of MMP9 in HUVECs thereby exerting a migration, invasion, and tube formation inhibitory effect. The stimulation of HIF-1α regulates the expression of angiogenic genes such as VEGF. As the crucial molecule in Wnt/β-catenin pathway, β-catenin, has a pivotal effect on cell migration and angiogenesis when receiving upstream gene regulation including HIF-1α [40,45]. However, our results suggested that BTDE had no effect on the expression of these molecules on HUVECs, which was different from BDDE, a bromophenol through inhibiting VEGF signaling plays an anti-angiogenesis effect [25]. Nevertheless, both of them were found to reduce HUVECs migration and tube formation, indicating that BTDE exerts the anti-angiogenesis effect through other signaling and the mechanisms still needs to be further explored.

In addition to the endothelial cell-dependent angiogenesis, another important factor for tumor blood supply is the diverse tumor vessels composition [46]. Vasculogenic mimicry is the microcirculation channel consisting of the aggressive tumor cells connection and extracellular matrix [9]. Many studies have confirmed the existence of vasculogenic mimicry in solid tumors such as melanoma, ovarian cancer, and lung cancer, and the poor prognosis of advanced cancer patients is significantly related with tumor vascular mimicry [47]. All these indicate that targeting vasculogenic mimicry therapy is a crucial strategy in tumor treatment. In our study, it is noteworthy that BTDE had a significant migration inhibitory effect on A549, H1975 cells. Moreover, BTDE also restrained the vasculogenic mimicry formation ability of A549 while had no impact on HIF-1α, β-catenin, VEGF, and the downstream signaling molecules. BTDE may target on other possible mechanisms such as EMT process [16], VE-cadherin [48], and wnt5a which are involved in the activation of Wnt signaling, and participated in cells proliferation, migration, adhesion, and vascularization [49,50]. The clear mechanism by which BTDE works remains to be further explored.

The anti-angiogenic activity of cancer chemopreventive agents is usually through inhibiting or retarding the development of tumor blood vessels [51]. For example, clinical antioxidant compound Nacetyl-L-cysteine is able to restrain the migration ability of melanoma cells, and to suppress endothelial cell invasion through inhibiting MMPs activity [52,53]. Similarly, our previous study has also showed the antioxidant effect of BTDE on HaCaT cells [28]. In the present studies, we revealed that BTDE inhibited the migration, invasion, and vasculogenic mimicry of A549 cells, as well as reducing HUVECs tube formation, MMP9 activity, and zebrafish embryo angiogenesis. All these investigations showed BTDE had valid anti-angiogenesis function and could be developed as potential angioprevention agent.

## 4. Materials and Methods

### 4.1. Drugs and Reagents

BTDE (purity > 98%) was provided by School of Medicine and Pharmacy. Antibodies against HIF-1α were purchased from Affinity (Canal Fulton, OH, USA), against β-catenin were purchased from Santa Cruz Biotechnology (Dallas, TX, USA), against VEGF, GAPDH, Tubulin were purchased from HuaAn Biotechnology (Hangzhou, China). Antibodies against AKT, p-AKT, ERK, p-ERK were purchased from Cell Signaling Technology (Boston, MA, USA). Transwell inserts (8 μm) were purchased from Corning company (Corning Costar, Cambridge, MA, USA). Matrigel was the product of BD company (Becton Dickinson, Bedford, MA, USA).

### 4.2. Cell Lines and Cell Culture

Human umbilical vein endothelial cells line HUVECs was from American Type Culture Collection (Gaithersburg, Maryland), human lung cancer cell lines A549 and H1975 were from Shanghai Cell Bank (Shanghai, China), Chinese Academy of Science. HUVECs was cultured in Dulbecco’s modified Eagle’s medium (DMEM, GIBCO, Grand Island, NY, USA). Human lung cancer cell lines A549 and H1975 were kept in RPMI-1640 medium (Gibco-BRL). All mediums were supplemented with 10% fetal bovine serum (FBS) and 1% penicillin/streptomycin. Cells were cultivated in a humidified incubator containing 5% CO_2_ at 37 °C.

### 4.3. Cell Viability Assay

The proliferation effects of BTDE on differentiated HUVECs, A549, and H1975 cells were determined by 3-(4,5-dimethylthiazol-2-yl)-2,5-diphenyl-2H-tetrazolium bromide (MTT) assay. In brief, cells were plated in 96-well plate overnight to adhere, then 0−20 μM BTDE were administrated and incubated for 36 or 48 h. Total of 5 mg/mL MTT solution (20 μL per well) was added and incubated for another 4 h at 37 °C, discarding the supernatant and using dimethyl sulfoxide (DMSO) to dissolve products for 10 min at 37 °C. Microplate reader (BioTek, Winooski, VT, USA) was used to measure the 96-well plate at 570 nm, and the cell viability (%) was calculated by OD values.

### 4.4. Scratch-Wound Cell Migration Assay

HUVECs, A549, and H1975 were plated in 96-well plate and cultivated to reach 90% confluence. Then scratch-wounds were made by 10 μL pipette tip. After washing twice with PBS, the cells were treated with fresh DMEM or RPMI-1640 (1% FBS) with 0−10 μM BTDE. After incubation for 36 or 72 h, images of wound in each well were recorded by inverted microscope (NIB-100, Novel Optics, Ningbo, China, original magnification, 4×). Image J was used to measure the area of each wound, and the migration rate was calculated as follows:

Wound healing area (%) = (Area _0 h_ − Area _t h_)/Area _0 h_ × 100%

### 4.5. Transwell Migration and Invasion Assay

Transwell chamber with 8 μm pore size was used to evaluate the migration of cells. Briefly, differentiated HUVECs, A549, and H1975 cells suspended in 1% FBS medium with 0−10 μM BTDE were seeded into the upper chamber of transwell 24-well plates. Then the lower chamber was added with complete medium and different 0−10 μM BTDE. After treatment for 24 h, the chambers were fixed with methanol, stained with 0.1% crystal violet, and the upper surface of the membrane containing non-migrating cells was gently wiped off with cotton swab. Random visual fields were calculated using inverted microscope (NIB-100, CHN, original magnification, 10×), chambers were decolorized with 33% acetic acid, then each group of decolorizing solutions was transferred to a new 96-well plate. A microplate reader (BioTek, Winooski, VT, USA) was used to measure OD values of the plate at 570 nm.

To investigate the invasion ability of HUVECs and A549, transwell invasion assay was conducted similarly with transwell migration assay except that the upper side of the chambers was spread with diluted matrigel (200 μg/mL).

### 4.6. HUVECs Tube Formation and A549 Vasculogenic Mimicry Assay

The ability of HUVECs or A549 to form capillary-like structures when treated with 0−10 μM BTDE was measured on matrigel. Briefly, pre-cooled matrigel was layered in 96-well plate and allowed to solidify at 37 °C for 45 min. HUVECs or A549 that has been treated with BTDE for 24 h was seeded on matrigel, after 20 h incubation for HUVECs or 30 h for A549, tube structure was recorded by inverted microscope (NIB-100, Novel Optics, Ningbo, China, original magnification, 4×) from randomly chosen fields. For investigating the effect of BTDE on performed vascular tubes, same concentrations of BTDE were added with HUVECs or A549 after tubes had already formed for 8 or 6 h, and incubated for another 6 h for HUVECs and 20 h for A549. Total length of tubes was measured by Image J software (version 1.48, National Institutes of Health, Rockville Pike, Maryland).

### 4.7. Zebrafish Embryo Assay

For intersegmental vessel formation assay, Tg (flk1: EGFP) zebrafish embryos were generated by natural pairwise mating. Healthy, hatched zebrafish were picked out at 16 h post-fertilization and distributed into a 24-well plate (10 embryos per well). Embryos were treated with 0−10 μM BTDE for 24 h at 28.5 °C and then observed for intersegmental blood vessel (ISV) under inverted fluorescence microscope (DM6000, Leica, Wetzlar, Germany). Vessel growth was measured by Image J software.

For toxicity assay, zebrafish embryos were picked out at 4 h post-fertilization and distributed into a 24-well plate (about 17 embryos per well). Embryos were treated with 0−20 μM BTDE for 24 h at 28.5 °C and then observed for morphologic changes under stereo microscope (SMZ645, Nikon, Tokyo, Japan). The deformity and mortality rates were recorded.

### 4.8. Gelatin Zymography Assay

Gelatin zymography assay was used to determine the activity of MMP9. HUVECs was treated with different concentrations of BTDE in serum free DMEM for 24 h, then culture supernatants were collected and centrifuged at 1200 rpm for 5 min, and then 12,000 rpm for 5 min to remove cellular components. Proteins existed in supernatants and were separated by 8% SDS-PAGE containing 1 mg/mL gelatin under non-reducing condition and then subjected to electrophoresis. Gels were washed twice for 40 min each time in washing buffer (2.5% Triton X-100/50 mM Tris/5 mM CaCl_2_/1 μM ZnCl_2_, pH 7.6), and washed twice for 40 min each time in rinse buffer (50 mM Tris/5 mM CaCl_2_/1 μM ZnCl_2_, pH 7.6), then incubated 48 h at 37 °C in renaturation solution containing 50 mM Tris/0.15 M NaCl/5 mM CaCl_2_/1 μM ZnCl_2_ and 0.02% Brij-35, pH 7.6). Gels were finally stained with 0.05% Coomassie Blue R250 for 30 min and decolorized with decolorizing liquid (10% acetic acid and 30% methanol) until negative staining bands appear. Gels were recorded by Bio-Red Gel Imaging Analysis System (bio-rad GelDoc XR, Hercules, CA, USA).

### 4.9. Western Blotting

HUVECs and A549 were treated with different concentrations of BTDE (0−10 μM) for 24 h. Cells were centrifuged, then washed twice with PBS and lysed with loading buffer for 45 min in 4 °C. Then, the cell lysate was boiled for 10 min and stored at −80 °C. Protein samples were resolved by 8–10% SDS-PAGE, transferred to nitrocellulose filter membranes (Millipore, Billerica, MA, USA). The nitrocellulose filter membranes were blocked with 5% skimmed milk and then incubated with the primary antibodies overnight at 4 °C. Subsequently, the membranes were incubated with HRP-secondary antibody at 25 °C for 1 h. Finally, the image was detected by Tanon 5200 (Tanon, Beijing, China).

### 4.10. Statistical Analysis

Statistical analysis was performed using one-way ANOVA with Tukey’s post-hoc test, and values were expressed as mean ± SD. Differences of *p* < 0.05 were considered statistically significant.

## 5. Conclusions

The present study illustrated for the first time that BTDE inhibited HUVECs migration, invasion, tube formation, and the activity of MMP9 in vitro. In zebrafish embryo, BTDE also restrained the growth of zebrafish embryo intersegmental blood vessel. In addition, BTDE suppressed A549, H1975 migration and A549 invasion, as well as the vasculogenic mimicry of A549 cells. These results definitely revealed that marine bromophenol compound BTDE was a potential agent for future cancer therapy due to its valid anti-angiogenesis effect.

## Figures and Tables

**Table 1:** The effect of BTDE on zebrafish embryo deformity and mortality.

BTDE (μM)	Number of Embryos	Number of Deformities	Deformity Rate (%)	Number of Mortalities	Mortality Rate (%)
0	71	2	2.8	0	0
2.5	70	1	1.4	0	0
5	71	1	1.4	1	1.4
10	70	0	0	2	2.8
20	73	1	1.4	0	0
